# Antimicrobial activity and mechanism of action of Nu-3, a protonated modified nucleotide

**DOI:** 10.1186/1476-0711-10-1

**Published:** 2011-01-14

**Authors:** Shanping Cao, Lun-Quan Sun, Ming Wang

**Affiliations:** 1 College of Veterinary Medicine, China Agricultural University No.2 Yuanmingyuan West Road, Beijing, 100193, China; 2 Xiangya Hospital and Center for Molecular Medicine, Central South University, Changsha, 410078, China

## Abstract

**Background:**

"Nubiotics" are synthetic oligonucleotides and nucleotides with nuclease-resistant backbones, and are fully protonated for enhanced ability to be taken up by bacterial cells. Nu-3 [butyl-phosphate-5'-thymidine-3'-phosphate-butyl], one of the family members of Nubiotics was efficacious in the treatment of burn-wound infections by *Pseudomonas aeruginosa *in mice. Subsequent studies revealed that Nu-3 had a favorable toxicological profile for use as a pharmaceutical agent. This study evaluated the antibacterial activity of Nu-3 in vitro and its efficacy as a topical antibiotic. In addition, we investigated the possible mechanisms of Nu-3 action at the levels of DNA synthesis and bacterial membrane changes.

**Methods:**

Antimicrobial minimum inhibitory concentrations (MIC) experiments with Nu-3 and controls were measured against a range of Gram-positive and Gram-negative bacteria, including some hospital isolates according to Clinical and Laboratory Standards Institute (CLSI) guidelines. Analysis of the killing kinetics of Nu-3 was also performed against two strains (*Staphylococcus aureus *cvcc 2248 and *Pseudomonas aeruginosa *cvcc 5668). The mouse skin suture-wound infection model was used to evaluate the antibacterial activity of Nu-3. We used a 5-Bromo-2'-deoxy-uridine Labeling and Detection Kit III (Roche, Switzerland) to analyze DNA replication in bacteria according to the manufacturer's instruction. The BacLight™ Bacterial Membrane Potential Kit (Invitrogen) was used to measure the bacterial membrane potential in *S. aureus*.

**Results:**

Nu-3 had a wide antibacterial spectrum to Gram-positive, Gram-negative and some resistant bacteria. The MIC values of Nu-3 against all tested MRSA and MSSA were roughly in a same range while MICs of Oxacillin and Vancomycin varied between the bacteria tested. In the mouse model of skin wound infection study, the treatment with 5% Nu-3 glycerine solution also showed comparable therapeutic effects to Ciprofloxacin Hydrochloride Ointment. While Nu-3 had no effect on DNA synthesis of the tested bacteria as demonstrated in a BrdU assay, it could cause bacterial cell membrane depolarization, as measured using a BacLight™ Bacterial Membrane Potential Kit.

**Conclusions:**

These results provide additional experimental data that are consistent with the hypothesis that Nu-3 represents a new class of antibacterial agents for treating topical infections and acts via a different mechanism from conventional antibiotics.

## Background

Microorganisms resistant to multiple anti-infective agents have increased around the world [1]. Bacterial resistance in community settings has also become a great concern and Methicillin-resistant *Staphylococcus aureus *(MRSA) is a frequent cause of health care- and community-associated infections. This is especially true in countries with limited resources [2,3]. Ecological pressure derived from the use of antimicrobial agents is the main driving force for the emergence of the resistance. Heroic efforts has been made both in academic institutions and pharmaceutical industries in response to the increasing medical need and to increasing resistance of bacteria to both individual classes of antibiotics and especially across different classes [4]. Unfortunately, the development of new antibiotics has not kept pace with the increase in prevalence of multi-drug resistant pathogens. To reduce the human mortality and morbidity associated with the infectious diseases caused by drug-resistant bacterial pathogens, there is a compelling need to develop new therapeutic agents that are effective against drug-resistant mutants [5].

**Figure 1:**
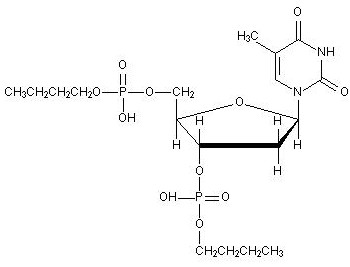
Chemical structure of Nu-3

"Nubiotics" are synthetic oligonucleotides and nucleotides with nuclease-resistant backbones, and are fully protonated for enhanced ability to be taken up by bacterial cells. Nu-3 [butyl-phosphate-5'-thymidine-3'-phosphate-butyl], one of the family members of Nubiotics, is a fully protonated and protected deoxynucleotide and its chemical structure is shown in Figure 1. Previous studies demonstrated that Nu-3 was efficacious in the treatment of burn-wound infections by *Pseudomonas aeruginosa *in mice [6]. Subsequent studies revealed that there was no chronic toxicity to healthy mouse skin when Nu-3 was applied topically [7], indicating that Nu-3 has a favorable toxicological profile for use as a topical antibiotic. In order to further explore how wide the spectrum of Nu-3 antibacterial activity is, MIC and time-kill experiments were performed with a wide range of Gram-positive and Gram-negative bacteria, including some hospital isolates of meticillin-resistent *Staphylococcus aureus *(MRSA) and meticillin-susceptible *Staphylococcus aureus *(MSSA). In addition, we evaluated Nu-3 antibacterial activity in a mouse skin suture-wound infection model against both the gram positive (*Staphylococcus aureus*) and the gram negative (*Pseudomonas aeruginosa*) infections. We also investigated the possible mechanisms of Nu-3 action at the levels of DNA synthesis and bacterial membrane changes.

## Materials and methods

### Animals

BALB/c mice (females, 18-20 g, 8 weeks, from Vitalriver Animal Center, Beijing), along with original breeding pairs purchased from Charles River (Canada), were housed under normal conditions for 5-7 days before being used for experiments. The use and care of the animals were performed according to the Regulations for the Administration of Affairs Concerning Experimental Animals in China (11/14/1988).

### Bacteria

The test organisms (*Brucella abortus*, *Burkholderia mallei, Burkholderia pseudomallei, Bacillus anthracis, Francisella tularensis, Yersinia pestis*) for the MIC assays were recent, predominantly ocular, isolates of United States origin. Five MRSA isolates consisted of non-consecutive, non-duplicate clinical isolates collected from Stanford University. *Staphylococcus aureus *(cvcc 2248) and *Pseudomonas aeruginosa *(cvcc 5668) were purchased from China Institute of Veterinary Drug Control (CIVDC). These two isolates were collected from the dermatology department of Peking Union Medical College Hospital. Quality control strains were *S. aureus *ATCC 25923, *P. aeruginosa *ATCC 27853, MSSA ATCC 29213, MRSA ATCC 33591 and *S. aureus *ATCC 700699 GISA. The bacterium was initially cultured on trypticase soy broth (TSB), divided into aliquots, and frozen at -80°C. Prior to use, aliquots were thawed and diluted serially in sterile phosphate-buffered saline (PBS). To ensure viability and virulence, aliquots of the bacteria were periodically re-amplified in TSB and colonies determined on trypticase soy agar (TSA) plates.

### Antimicrobial agents

Nu-3 was synthesized and purified at Oligos Etc., Inc. (Oregon) with 99.5% purity (HPLC). The concentration of the agent was determined based on OD260 reading. Ciprofloxacin, Oxacillin and Vancomycin were purchased from Sigma (St. Louis, MO, USA). Ciprofloxacin Hydrochloride Ointment (30 mg active ingredient per 10 grams) was purchased from TongRenTang Drug Store (Beijing).

### Determination of minimal inhibitory concentration (MIC)

MICs were determined using a standard 2-fold micro-dilution method in Mueller-Hinton broth (Difco) and antibacterial susceptibilities were reported using CLSI interpretive criteria [8-10]. The highest drug concentration of Nu-3 tested in this assay was 2000 A/ml (117.6 mg/ml). To each well of a 96 well microtiter plate, 30 μl of the different dilutions and 170 μl of MHB medium containing 10^6 ^bacteria/ml were added. Plates containing *Burkholderia, Brucella *and *Francisella *were incubated at 37°C with 5% CO_2_. The incubation time for *Brucella *was 48 hours while *Francisella *and *Yersinia *were incubated a total of >72 hours. The remaining strains of *Bacillus*, *Staphylococcus *and *Pseudomonas *were incubated for 24 hours at 37°C. Plates were scored and numerical values were given to represent the extent of growth. We scored "3" for maximum growth, "2" for moderate growth, "1" for minimum growth and "0" for no growth. The MIC was defined as the minimum amount of Nu-3 that resulted in no visible growth after incubation.

### Time-progression curve for bacterial killing

The determination of the killing curve was carried out as described previously with the following modifications [11]. Briefly, aliquots of the bacterial suspension were removed from the -80°C freezer and thawed at room temperature.10 ul of the bacterial suspension was inoculated in a tube with 5 ml TSB (BD) and then incubated at 35°C with gentle shaking. The doses of Nu-3 and Ciprofloxacin were added to the culture tubes at 2 × MIC, 1 × MIC (final concentration). Rates of killing were determined by measuring the reduction in the number of viable bacteria (ΔCFU/ml log_10_) at 0, 2, 4, 6, 12, and 24 h at the fixed concentrations of Nu-3. Experiments were performed in duplicate. If plates contained fewer than 30 CFU/ml, then the number of colonies was considered to be below the limit of quantization [11]. Samples of cultures containing Nu-3 were diluted at least 10-fold to minimize drug carryover to the TSA (BD) plates.

### Mouse wound infection model

The establishment of the skin suture-wound model was carried out as described previously [12]. Briefly, sterile silk sutures were cut into 10-cm lengths and threaded onto sterile surgical needles, followed by being soaked for 45 min in undiluted broth cultures of the *S. aureus *2248 and *P. aeruginosa *5668 that had been incubated at 35°C for 8 h. The sutures with sterile surgical needles were removed aseptically, dried on sterile filter paper at 4°C until the animals were prepared for surgery. Animals were anesthetized with 50 mg/kg Zoletil-50 (Virbac, France) intramuscularly. The fur on the back and flanks was clipped, and the skin was swabbed with 70% ethanol. Before inoculating the suture, the knot was made on one side of the suture. By using the threaded needle, a 1-cm length of inoculated suture was inserted under the skin of the mid-back and secured by knotting on the other side of the suture. An incision was made along the length of the suture between the two knots. The depth of the incision did not reach into the panniculus carnosus. One wound was created per animal. To count the bacteria carried on the sutures, 1-cm length of each suture was vortexed in 1 ml of TSB (BD) for 15 min for the *S. aureus *2248 and *P. aeruginosa *5668. The suspensions were serially diluted, and 10-ul of each dilution was plated onto TSA (BD) in triplicate, and then the plates were incubated for 24 h. The numbers of organisms per centimeter on each suture were calculated.

### Nu-3 treatment of animals

To treat the infected mouse, 5% (50 mg Nu-3 in 1 ml 60% glycerin solutions) and 1% (10 mg Nu-3 in 1 ml 60% glycerin solutions) of Nu-3 glycerin solutions were made in sterile water. Nu-3 glycerin solutions (100 μl) and Ciprofloxacin Hydrochloride Ointment (0.2 g) were applied topically onto the infection sites. Infected control mice without drug treatment were given an equal volume of phosphate buffered saline (PBS) and glycerin water solutions. Treatment was initiated 4 h after surgery and a second application was made 8 h later. The treatment was continued three times daily for a further 3 days. At 5 days post treatment, the mice were euthanized by zoletil-50 overdose. A 1-by-1-cm area of skin, including the wound, was excised and homogenized in 1 ml of TSB in glass tissue grinders. The homogenates were serially diluted, and the organisms were numerated as previously described.

### BrdU incorporation Assay

We used a 5-Bromo-2'-deoxy-uridine Labeling and Detection Kit III (Roche, Switzerland) to analyze DNA replication in bacteria according to the manufacturer's instruction. In brief, Nu-3 in a range of concentrations was added to the testing bacteria. 100 ul per well of the treated bacteria suspension (ca. 6 × 10^6 ^CFU/ml for *S. aureus *2248) was added into a 96-well microplate, and 10 ul BrdU labeling solution added to each well, then incubated for 8 h at 37°C. The suspension was spun for 10 min at 300 × g in a microcentrifuge (Eppendorf, Germany) and the labeling medium was carefully removed. The bacteria were allowed to dry at the bottom of the microplate for 2 h at 60°C and then fixed with 200 ul pre-cooled fixative per well for 2 h at -20°C. After removal of the fixative, the plate was washed 3 times with 250 ul PBS, followed by adding 100 ul of nucleases working solution per well and incubating the mixture for 30 min at 37°C water bath. The nucleases working solution was then removed and washed 3 times with 250 ul PBS. To the each well, 100 ul anti-BrdU-POD working solution was added and incubated for 30 min at 37°C. The antibody conjugate was removed by 3 washes with 250 ul washing buffer and 100 ul peroxidase substrate with substrate enhancer per well was added and incubated at room temperature for 15 min. Finally, extinction of the samples was measured in a microplate reader at 405 nm with a reference wavelength at 490 nm as the rate of incorporation of BrdU into bacterial DNA. In the study, we used Ciprofloxacin as a positive control since fluoroquinolones acts by interacting with type II topoisomerases (DNA gyrase and topoisomerases IV) to inhibit DNA synthesis [13].

### Measurement of bacterial membrane potential

The BacLight™ Bacterial Membrane Potential Kit (Invitrogen) was used to measure the bacterial membrane potential in *S. aureus*. Diethyloxacarbocyanine (DiOC_2_) exhibits green fluorescence in all bacterial cells, but the fluorescence shifts toward red emission as the dye molecules self associate at - higher cytosolic concentrations caused by larger membrane potentials. Proton ionophores such as carbonyl cyanide m-chlorophenylhydrazone (CCCP) could destroy membrane potential by eliminating the proton gradient, thus it was used as a positive control in the study. The ratio of red to green fluorescence provides a measure of membrane potential that is largely independent of cell size, with a low coefficient of variation (CV) [14]. Bacteria was grown in TSB (BD) overnight and diluted in filtered PBS to approximately 1 × 10^6 ^CFU/ml. Aliquots of 1 ml bacterial suspension were added into flow cytometry tubes for staining treatments with a depolarized control and an unstained control. 100 μl of Nu-3 in a range of concentrations was added to each sample tube, and 10 μl of 500 uM CCCP to the depolarized control sample. All samples were then incubated for 30 min at 4°C, followed by adding 5 μl of 3 mM DiOC_2 _to each flow cytometry tube, mixing well and incubating at room temperature for 15 minutes. Stained bacteria were assayed in a flow cytometer (BD) with a laser emitting at 488 nm. Fluorescence was collected in the green and red channels ("GC" and "RC"); the unstained control sample was used to locate bacterial populations in the forward and side scatter channels. The bacteria population was gated using forward versus side scatter and fluorescence photomultiplier tube voltages were adjusted such that the green and red MFI values were approximately equal without compensation. While the relative amount of red and green fluorescence intensity varied with respect to cell size and aggregation, the ratio of red to green florescence intensity can be used as a size-independent indicator of membrane potential. For a dot plot of red versus green fluorescence, the regions around the populations of interest were set and red and green MFI values for each recorded. The change of the membrane potential was expressed as MFI of the red population divided by the green population MFI.

### Statistical analysis

Statistical analysis of the bacterial counts between control and test groups was carried using one-way ANOVA. Significance was evaluated at p-value of 0.05.

## Results and discussion

### Antibacterial activity of Nu-3 in vitro

To determine the spectrum of Nu-3 antibacterial activity, antimicrobial minimum inhibitory concentrations (MIC) of Nu-3 and controls against a range of Gram-positive and Gram-negative bacteria, including some hospital isolates of meticillin-resistent *Staphylococcus aureus *(MRSA) and meticillin-susceptible *Staphylococcus aureus *(MSSA), were performed. The results showed that Nu-3 had a wide antibacterial spectrum to Gram-positive, Gram-negative and some resistant bacteria (Table 1). The MIC values of Nu-3 against all tested MRSA isolates were roughly in a same range while MICs of Oxacillin and Vancomycin varied between the bacteria tested. These data suggested that Nu-3 may act in a different mechanism from the conventional antibiotics.

**Table 1:** Minimum inhibitory concentrations of Nu-3 (mg/ml) and antibiotic controls (μg/ml) (ciprofloxacin, oxacillin and vancomycin).

	MIC			
	
Strains	Nu-3 (mg/ml)	Ciprofloxacin (μg/ml)	Oxacillin (μg/ml)	Vancomycin (μg/ml)
*Brucella abortus*	>2.2	1	-	-
*Burkholderia mallei*	>4.4	1	-	-
*Burkholderia pseudomallei*	4.4	1	-	-
*Bacillus anthracis *(Sterne)	2.2	0.08	-	-
*Francisella tularensis*	2.2	0.08	-	-
*Yersinia pestis*	4.4	0.16	-	-
*Staphylococcus aureus cvcc 2248*	4.4	8	-	-
*Pseudomonas aeruginosa cvcc 5668*	8.8	4	-	-
MSSA ATCC 29213	8.8	-	0.4	1
MRSA ATCC 33591	>4.4	-	256	2
*S. aureus *ATCC 700699 GISA	8.8	-	>32	32
Stanford University MRSA Clinical Isolate #1	>4.4	-	256	2
Stanford University MRSA Clinical Isolate #2	8.8	-	128	1
Stanford University MRSA Clinical Isolate #3	8.8	-	>256	4
Stanford University MRSA Clinical Isolate #4	8.8	-	32	1
Stanford University MRSA Clinical Isolate #5	8.8	-	32	1

MRSA: meticillin-resistant Staphylococcus aureus.

**Figure 2:**
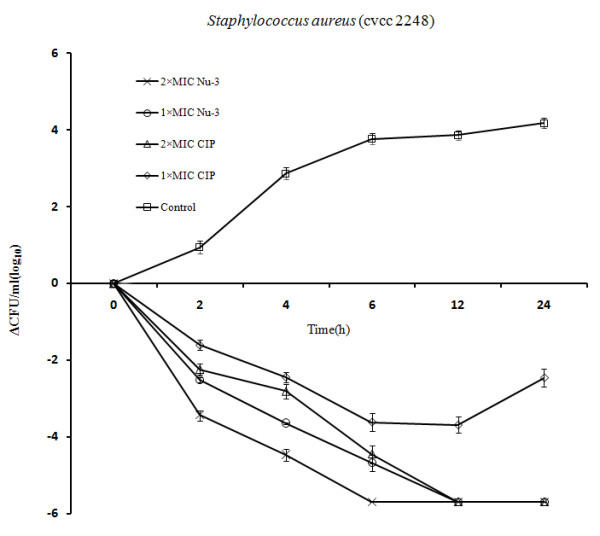
**Time-progression curves of killing S. aureus cvcc 2248 in different concentrations of Nu-3 (open circles: 1 × MIC; cross: 2 × MIC) and ciprofloxacin (CIP) (open diamonds: 1 × MIC; open triangle: 2 × MIC)**. Open squares: no-treatment growth control. The average values ± SD were used in the figure.

**Figure 3:**
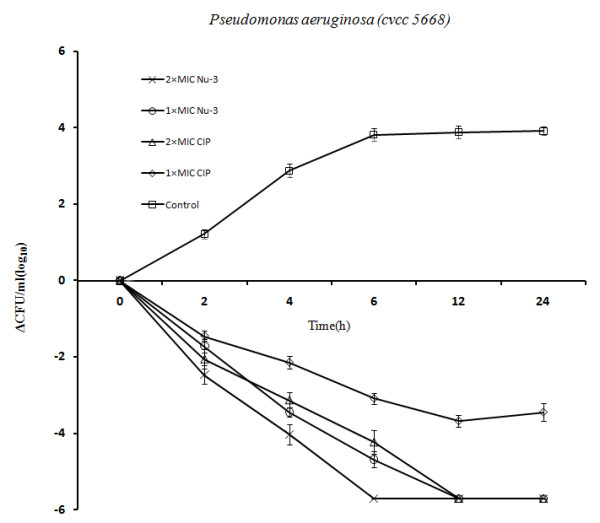
**Time-progression curves of killing *P. aeruginosa cvcc 5668 *in different concentrations of Nu-3 (open circles: 1 × MIC; cross: 2 × MIC) and ciprofloxacin (CIP) (open diamonds: 1 × MIC; open triangle: 2 × MIC)**. Open squares: no-treatment growth control. The average values ± SD were used in the figure.

Time-kill assays were conducted for *Staphylococcus aureus *2248 and *Pseudomonas aeruginosa *5668 in concentrations of 2 × MIC, and 1 × MIC. Figure 2 and Figure 3 showed a concentration-dependent killing of *Staphylococcus aureus *2248 and *Pseudomonas aeruginosa *5668 at 2, 4, 6, 12 and 24 h after the addition of Nu-3 and Ciprofloxacin. It revealed that those two strains had a similar time-kill profile with Nu-3 killing *S. aureus *2248 and *P. aeruginosa *5668 at a very fast rate. In concentration-dependent killing of *S. aureus *2248, a 1000-fold reduction in CFU was observed after 2 h for Nu-3 (2 × MIC) and a 100-fold reduction for ciprofloxacin (2 × MIC). In the concentration-dependent killing of *P. aeruginosa *5668, a 1000-fold reduction in CFU was observed after 4 h for Nu-3 and ciprofloxacin (2 × MIC). When a dose of 1 × MIC of Nu-3 and ciprofloxacin was used, a 1000-fold reduction in CFU was observed at 4 h for Nu-3, but it needed 12 h to reach the same level of reduction in CFU for ciprofloxacin. For Nu-3, the CFUs from *S. aureus *2248 and *P. aeruginosa *5668 were reduced to zero within 6 h at both 2 × MIC and 12 h at 1 × MIC while for Ciprofloxacin, the time to zero in CFU was 12 h (2 × MIC) for both strains. These data suggest that Nu-3 can kill bacteria at a faster rate than ciprofloxacin at concentrations of both 2 × MIC and 1 × MIC.

### Therapeutic efficacy of Nu-3 in mouse wound model

To evaluate potency of Nu-3 in vivo, the skin suture-wound model was used to topically test the antibacterial activity of Nu-3 [12]. First, we examined if Nu-3 could treat the wound infection in a dose-response manner. As shown in Table 2, Nu-3 glyerine solution could inhibit the growth of both *P. aeruginosa *and *S. aureus *in vivo. This inhibitory effect showed a dose-response with 1 log (1% Nu-3) and 2 log (5% Nu-3) reduction in CFU values in comparison with the untreated control (P < 0.01 and P < 0.001 respectively). We then compared Nu-3 with Ciprofloxacin Hydrochloride Ointment in their effect on the bacterial growth. Both Nu-3 and Ciprofloxacin gel showed a similar therapeutic effect in the skin suture-wound model, as compared with the untreated animals (P < 0.001) (Table 3). Nu-3 in glycerin formulation appeared to be slightly better than that in saline although the difference was not statistically significant.

**Table 2:** Dose-response of Glycerin-Nu-3 solution in inhibition of *P. aeruginosa cvcc 5668 *and *S. aureus cvcc 2248 *growth in mouse suture-wound model.

	**Mean bacterial counts ± SD (Log10CFU/wound)**^**a**^	
	
Treatments	*P. aeruginosa cvcc5668*	*S. aureus cvcc2248*
Initial innoculation	4.66 ± 0.12	4.88 ± 0.56
Untreated	7.24 ± 0.30	7.18 ± 0.57
Glycerin solution	6.86 ± 0.19	7.04 ± 0.48
1% Nu-3 in Glycerin	6.73 ± 0.27*	6.34 ± 0.32*
5% Nu-3 in Glycerin	5.76 ± 0.42**	4.95 ± 0.21**

a, n = 6; * P < 0.01; **P < 0.001 in comparison with the untreated control. 5% Nu-3 is equivalent to 50 mg Nu-3 in 1 ml 60% glycerin solutions and 1% Nu-3 is 10 mg Nu-3 in 1 ml 60% glycerin solutions.

**Table 3:** Comparative study of Nu-3 against *P. aeruginosa cvcc 5668 *and *S. aureus cvcc 2248 *with Ciprofloxacin in mouse suture wound model.

Treatments	**Mean bacterial counts ± SD (Log10CFU/wound)**^**a**^	
	
	*P. aeruginosa cvcc5668*	*S. aureus cvcc2248*
Initial innoculation	4.93 ± 0.33	4.79 ± 0.31
Untreated	7.52 ± 0.57	7.64 ± 0.58
Glycerin solution	7.34 ± 0.27	7.47 ± 0.34
5% Nu-3 in saline	5.62 ± 0.61**	5.23 ± 0.5**
5% Nu-3 glycerin solution	5.07 ± 0.53**	4.51 ± 0.55**
Ciprofloxacin hydrochloride ointment	4.82 ± 0.58**	5.63 ± 0.31**

a, n = 6; ** P < 0.001 in comparison with the untreated control. 5% Nu-3 is equivalent to 50 mg Nu-3 in 1 ml 60% glycerin solutions and 1% Nu-3 is 10 mg Nu-3 in 1 ml 60% glycerin solutions.

The mouse model of skin wound infection was established by implanting contaminated sutures [15]. This model represents the secondary skin infections that may occur following damage by accidental trauma, and surgery. The skin is a milieu for controlled bacterial growth. Resident gram-positive bacteria include *Staphylococcus aureus *and *Streptococcus pyogenes *are notoriously pathogenic in the skin [16]. *Pseudomonas aeruginosa *is an opportunistic pathogen found along with other *Pseudomonas *spp. as part of the normal flora of the human skin [17]. When the host is immunocompromised, as in the case of a surgical wound, this opportunistic bacterium can quickly colonize and infects the wound site and then penetrates the blood capillaries of the affected tissues and thus may lead to bacteremia. Moreover, the virulence factors which the bacteria produced can lead to endotoxic shock. Once reaching the bacteremia or endotoxic shock phase, wound infections are generally beyond treatment by conventional antibiotic therapy. Therefore, it is imperative that effective antibiotic treatment be administered at an early stage of infection to be efficacious. In the present study, two treatments were applied at 4 h and 8 h after surgery to eliminate the bacteria at an early stage. During 5 days of the observation, the bacterial count from each wound increased to 10^7 ^at the 5^th ^day from an initial inoculation with 10^4 ^bacteria of either strains in the untreated and placebo groups. Topical treatment with 1-5% Nu-3 glycerine solution significantly reduced the numbers of *P. aeruginosa *5668 and *S. aureus *2248 CFU after the treatment (Table 2 and 3). These data clearly show that the established topical treatment is effective in this model. The treatment with 5% Nu-3 glycerine solution also showed comparable therapeutic effects to Ciprofloxacin Hydrochloride Ointment. Therefore, Nu-3 may represent a promising externally applied agent for treating wound infection.

### Possible mechanisms of action of Nu-3

Modes of action for antimicrobials vary depending on their biochemical properties. The major mechanisms of action for antibiotics include inhibiting protein synthesis, nucleic acid synthesis and selectively disrupting bacterial metabolism. Chemically, Nu-3 is a modified nucleotide, which could potentially affect nucleic acid synthesis and function, and less likely interfere with other biological processes. Thus, we investigated if Nu-3 had any impact on nucleic acid synthesis by using BrdU incorporation assay. In addition, since Nu-3 is a protonated molecule, it is possible that Nu-3 may act through changing membrane polarity.

#### Effect of Nu-3 on DNA synthesis

**Figure 4:**
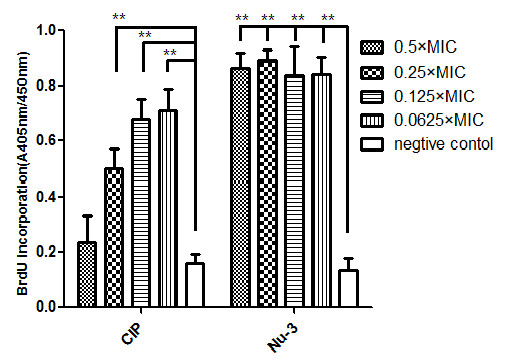
**Assays were performed in a 96-well microplate with triplicates for each sample**. Data were from three independent experiments and expressed as average ± SD. The concentration of Nu-3 and ciprofloxacin (CIP) in this assay were 0.5 × MIC, 0.25 × MIC, 0.125 × MIC and 0.0625 × MIC and the tested bacteria was S. aureus cvcc 2248. **P < 0.001 in comparison with the control.

The incorporation of 5-Bromo-2'-deoxy-uridine (BrdU) was monitored as a parameter for DNA synthesis and cellular proliferation. Cells that have incorporated BrdU into DNA can be easily detected using a monoclonal antibody against BrdU [18]. As shown in Figure 4, in a range of Nu-3 concentrations (0.5 × MIC, 0.25 × MIC, 0.125 × MIC and 0.0625 × MIC), the testing compound did not inhibit the incorporation of BrdU into the bacterial DNA as there were no significant differences in incorporation rate between each Nu-3 treated group, while ciprofloxacin as a positive control could inhibit DNA synthesis in the tested concentrations. The maximum concentration used in the study was 0.5 × MIC and the reason for this was that higher concentrations of Nu-3 may inhibit the growth of bacteria. The results clearly indicated that the Nu-3 action was not via interference to DNA synthesis. This can be easily comprehended in that Nu-3 is a modified thymidine with the phosphate-groups at both the 5'- and 3'-ends. (butyl-phosphate-5'-thymidine-3'-phosphate-butyl). It is unlikely that Nu-3 could compete with dTTP in the cellular environment to be incorporated into DNA during bacterial proliferation.

#### Effect of Nu-3 on bacterial membrane potential

**Figure 5:**
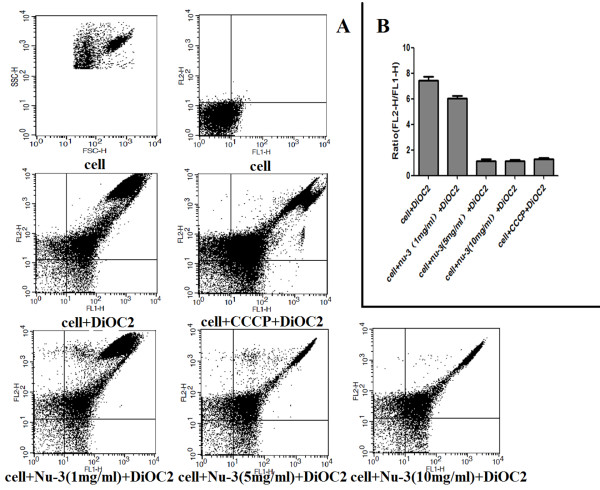
**Detection of the membrane potential in *S. aureus***. A: The red-versus-green fluorescence dot plot. B: The bar charts showed the red/green ratios in different treated groups. Ratiometric parameter was calculated using population mean fluorescence intensities from FACS data of counts vs FL1-H or FL2-H respectively. FL1-H: green fluorescence intensities; FL2-H: red fluorescence intensities.

In order to study the other potential mechanisms, the BacLight™ Bacterial Membrane Potential Kit was used to measure the bacterial membrane potential. It is known that changes in both green florescence (530 nm) and red fluorescence (600 nm) of the fluorescent dye DiOC_2 _(3) can reflect changes in membrane potential [19]. As shown in Figure 5A, 5 mg/ml and 10 mg/ml Nu-3 exhibited a similar absorption profile to the positive control CCCP, which is a proton ionophore and depolarized the cell membrane. Quantitatively, the ratiometric parameter (red/green) of both Nu-3 and CCCP treated cells decreased significantly as compared with untreated cells (Figure 5B). This result suggests that Nu-3 might inhibit bacterial growth via a membrane depolarization mechanism.

In metabolically active bacteria with intact cytoplasmic membranes, there is typically a difference of electrical potential across the membrane, with the interior negative by between 100 and 200 mV with respect to the exterior. This electrical potential is referred to as resting potential. A reduction in the magnitude of the membrane potential is referred to as electrical depolarization: an increase in the magnitude of membrane potential is referred to as electrical hyperpolarization. In this study, we, for the first time, show that Nu-3 could change the membrane potential that caused depolarization, possibly leading to the cell disruption, which was clearly shown in the bacterial cells using FACS (Figure 5) and fluorescence microscopy (data not shown). Therefore, this suggests that the membrane depolarization could be one of the mechanisms of antibacterial activity of Nu-3.

## Conclusions

In conclusion, the present study presented experimental evidence that supports the further development of Nu-3 as a novel antibiotic for topical uses in various applications, such as wound healing. With improved formulations and delivery means, for example, using liposome and nanoparticles, we hoped that better therapy can be provided for bacterial infections, particularly for existing drug-resistant strains.

## Competing interests

The authors declare that they have no competing interests.

## Authors' contributions

MW conceived and supervised the study. SC performed the experiments and wrote the manuscript in partial fulfillment for his PhD. LS provided intellectual support to SC and contributed to writing of the manuscript. All authors read and approved the final manuscript.
